# Exposure to gestational diabetes mellitus induces neuroinflammation, derangement of hippocampal neurons, and cognitive changes in rat offspring

**DOI:** 10.1186/s12974-017-0859-9

**Published:** 2017-04-07

**Authors:** Billy Vuong, Gary Odero, Stephanie Rozbacher, Mackenzie Stevenson, Stephanie M. Kereliuk, Troy J. Pereira, Vernon W. Dolinsky, Tiina M. Kauppinen

**Affiliations:** 1 Department of Pharmacology and Therapeutics, University of Manitoba, 753 McDermot Avenue, Winnipeg, Manitoba R3E 0T6 Canada; 2 Neuroscience Research Program, Kleysen Institute for Advanced Medicine, Health Sciences Center, SR434 – 710 William Avenue, Winnipeg, Manitoba R3E 0Z3 Canada; 3 The Children’s Hospital Research Institute of Manitoba, 601 John Buhler Research Centre, 715 McDermott Avenue, Winnipeg, Manitoba R3E 3P4 Canada

**Keywords:** Gestational diabetes mellitus, Hippocampus, Microglia, Neurodevelopmental disorder, Neuroinflammation, Recognition memory, Synapse

## Abstract

**Background:**

Birth cohort studies link gestational diabetes mellitus (GDM) with impaired cognitive performance in the offspring. However, the mechanisms involved are unknown. We tested the hypothesis that obesity-associated GDM induces chronic neuroinflammation and disturbs the development of neuronal circuitry resulting in impaired cognitive abilities in the offspring.

**Methods:**

In rats, GDM was induced by feeding dams a diet high in sucrose and fatty acids. Brains of neonatal (E20) and young adult (15-week-old) offspring of GDM and lean dams were analyzed by immunohistochemistry, cytokine assay, and western blotting. Young adult offspring of GDM and lean dams went also through cognitive assessment. Cultured microglial responses to elevated glucose and/or fatty acids levels were analyzed.

**Results:**

In rats, impaired recognition memory was observed in the offspring of GDM dams. GDM exposure combined with a postnatal high-fat and sucrose diet resulted in atypical inattentive behavior in the offspring. These cognitive changes correlated with reduced density and derangement of Cornu Ammonis 1 pyramidal neuronal layer, decreased hippocampal synaptic integrity, increased neuroinflammatory status, and reduced expression of CX3CR1, the microglial fractalkine receptor regulating microglial pro-inflammatory responses and synaptic pruning. Primary microglial cultures that were exposed to high concentrations of glucose and/or palmitate were transformed into an activated, amoeboid morphology with increased nitric oxide and superoxide production, and altered their cytokine release profile.

**Conclusions:**

These findings demonstrate that GDM stimulates microglial activation and chronic inflammatory responses in the brain of the offspring that persist into young adulthood. Reactive gliosis correlates positively with hippocampal synaptic decline and cognitive impairments. The elevated pro-inflammatory cytokine expression at the critical period of hippocampal synaptic maturation suggests that neuroinflammation might drive the synaptic and cognitive decline in the offspring of GDM dams. The importance of microglia in this process is supported by the reduced Cx3CR1 expression as an indication of the loss of microglial control of inflammatory responses and phagocytosis and synaptic pruning in GDM offspring.

## Background

Gestational diabetes mellitus (GDM) is a state of glucose intolerance and hyperglycemia with first onset during pregnancy and is the most common complication of pregnancy, affecting up to 10% of expectant mothers [1]. The prevalence of GDM is on the rise since more women are overweight or obese when they become pregnant. The combination of human cohort studies and research using rodent models with controlled pre- and postnatal conditions has demonstrated that diabetes during pregnancy during critical stages of development affects health outcomes in the offspring [2]. Several human cohort studies have linked diabetes during pregnancy with impaired cognitive abilities [3–6] and psychiatric disorders [7, 8] in the offspring. Similar correlations exist between poor quality early childhood diets and cognitive performance [9, 10]. Brain development is particularly sensitive to environmental influences during infancy [11]. Animal studies utilizing controlled conditions confirmed that maternal high-sugar intake caused deficits in cognitive functions in the offspring [12, 13]. However, the mechanisms that link GDM to the development of cognitive impairments in the offspring remain to be elucidated.

GDM is associated with mild but chronic systemic inflammation [14] and elevated circulating free fatty acids [15, 16]. Although the transfer of cytokines across the placenta from mother to fetus is restricted [17], maternal hyperglycemia and hyperlipidemia directly impact the fetus through the placental overproduction of pro-inflammatory cytokines [14, 18]. We have also recently shown that obesity-associated GDM promotes the production of the pro-inflammatory cytokine, interleukin (IL)-1β, and toll-like receptor activity, in the spleen cells of neonatal and 15-week-old offspring that may contribute to chronic low-grade systemic inflammation [19]. Cytokines can induce pro-inflammatory responses in the central nervous system (CNS) resulting in neuroinflammation, which includes the release of cytokines, chemokines, adhesion molecules, free radicals, and destructive enzymes. Activation of microglia, resident brain immune cells, is a central event and driving force in neuroinflammation [20]. Pro-inflammatory responses of activated microglia can jeopardize neurogenesis and promote neurodegeneration [21]. Microglia have an important role in neuroplasticity as they assist in the development and maintenance of neuronal networks via trophic factor release and controlled phagocytosis, which allows microglia to prune synapses and remodel neuronal transmission [22, 23]. Microglial pro-inflammatory responses [20] can also indirectly affect the neuronal vitality by changing the function of astroglial cells, which have supportive role in neuronal metabolism and modulation of synaptic transmission [24]. GDM-induced inflammation during embryogenesis could perturb microglial functions resulting in a limited ability to support the development of a healthy neuronal network that detrimentally influences brain development in the offspring.

We utilized a high-fat and sucrose (HFS) diet-induced GDM model [19, 25] to evaluate the effects of GDM exposure and postnatal diets on cognitive abilities and neuroinflammation in the offspring. In this GDM model, hyperglycemia is observed in mid-gestation and resolves after the dam litters out, thus mimicking some features of the clinical presentation of GDM in overweight and obese women [19, 25]. We determined that GDM exposure impaired recognition memory in the offspring and when combined with postnatal HFS diet, resulted in atypical inattentive behavior. These cognitive changes were associated with reduced density and derangement of the Cornu Ammonis 1 (CA1) pyramidal neuronal layer, decreased hippocampal synaptic integrity, and increased neuroinflammation. Exposure of primary cultures of microglia to elevated levels of glucose and/or fatty acids triggered pro-inflammatory responses capable of disturbing development of neuronal circuitry and functions, suggesting that gluco- and lipotoxicity contribute to microglial-mediated neuroinflammation.

## Methods

### Materials

Cell culture reagents were obtained from ThermoFisher, and all other reagents were from Sigma/Aldrich (St. Louis, MO) except where otherwise stated.

### Animal model

All animal procedures were approved by the Animal Welfare Committee of the University of Manitoba which follows the guidelines of the Canadian Council on Animal Care and the Council for International Organizations of Medical Sciences. Rats were given ad libitum access to food and water and were housed two per cage. As described previously [25], GDM was induced in female Sprague-Dawley rats using a high-fat and sucrose diet (HFS; 45% fat, 4.73 kcal/gm, Research Diets D12451; New Brunswick, NJ, USA) for 6 weeks prior mating, throughout gestation and lactation, while lean control dams were fed a low-fat diet (LF, 10% fat, 3.85 kcal/gm, Research Diets D12450B). All females were mated with lean males. The dams experienced increased weight gain during pregnancy and starting mid-gestation developed hyperinsulinemia and moderate hyperglycemia (Table 1). This model mimics clinical symptoms of GDM seen in mothers as well as their offspring that developed obesity and insulin resistance [2, 25]. Litters were randomly reduced to eight pups in order to avoid competition for food. The remaining offspring were weaned at 3 weeks of age, and males from each litter were randomly assigned to a LF or HFS postnatal diet. This created four experimental groups: offspring from LF-fed dams (“lean”) that were fed a LF or HFS postweaning diet and offspring from HFS-fed dams (“GDM”) that were fed a LF or HFS postweaning diet. All offspring underwent behavioral testing at 14 weeks of age and were sacrificed at 15 weeks of age, as young adults, when they were insulin resistant [25] but hyperglycemia was not observed (Table 2). In addition, neonatal (E20) offspring were collected for analysis.

**Table 1:** Maternal gestational blood glucose (at 3rd week of pregnancy) and weight gain during pregnancy

	Blood glucose (mmol/L)	Gestational weight gain (g)
Lean (4)	4.28 ± 0.38	133.6 ± 2.9
GDM (4)	6.40 ± 0.21*	155.5 ± 14.1*

*p ≤ 0.05 compared to Lean

**Table 2:** Offspring blood glucose (at 15 weeks of age) and weight gain (from weaning to 15 weeks of age)

	Blood glucose (mmol/L)	Postweaning weight gain (g)
Lean LF (6)	5.78 ± 0.55	557 ± 55.6
Lean HFS (8)	5.80 ± .0.22	568 ± 30.4
GDM LF (9)	5.92 ± 0.84	573 ± 63.8
GDM HFS (7)	5.43 ± 0.79	661 ± 57.7*

*p ≤ 0.05 compared to Lean LF

### Behavioral testing

Young adult rats had behavioral tests starting 1 week prior their sacrifice to analyze whether GDM and/or postweaning diet affected their cognition. The behavioral tests focus on analyzing the anxiety, attention, exploration, memory, and learning abilities. For the open field (OF) test, an arena with walls to prevent escape was used and the test was videotaped for later analysis to assess general activity level, anxiety, attention, and exploration tendency [26]. The rats were placed in OF (white square plexiglass chamber; L 75 cm × W 75 cm × H 75 cm, illuminated at 8–13 lx red light) for 10 min. Locomotor activity, rearing, and the amount of time spent in the center area of the OF were calculated. The novel object recognition (NOR) test of recognition memory which is affected by hippocampal and perirhinal cortex damage [27–31] was performed after the OF test. On the first day, rats were placed back to the testing arena which had now two identical objects. Rats were allowed to explore the objects freely for a 10-min training session. On the second day, rats were placed back into the testing arena for the 10-min test session, during which they were presented with one of the objects used during training (familiar object) and with a novel, unfamiliar object of different shape and texture. Object locations were kept constant during training and test sessions for any given rat. Arenas and objects were cleaned with 70% ethanol between each rat. Frequency of object interactions and time spent exploring each object was recorded with a HVS Image 2100 Plus video tracking system software (HVS Image Ltd., Twickenham, Middlesex, UK). Frequency of object interactions during the first 5 min was used for analyses.

### Brain immunostaining and cytokine measurements

Immunostaining was performed on 4% formaldehyde fixed 30-μm coronal sections with anti-NeuN (Chemicon international, 1:500), anti-synaptophysin (1:500), anti-Iba1 (Wako, 1:500), and anti-GFAP (EMD Millipore, 1:500) followed by Dylight 488 or 594 conjugated anti-IgG (Thermofisher, 1:500) as described previously [32]. ImageJ program (NIH) was used to measure hippocampal neuronal CA1 layer thickness, and synaptophysin and GFAP expressions were determined by the mean optical density in the designated, uniform-sized regions of interest [32, 33]. Microglial morphology identification was based on assessment of cell soma and processes (Table 3). Values were measured on a minimum of three matching hippocampal regions from each rat, background values were subtracted, and the resulting values were averaged to give one value per rat.

**Table 3:** Microglial morphology analysis criteria

	Ramified	Hypertrophic	Amoeboid
Cell soma	Small oval shaped	Enlarged	Enlarged
Processes	Numerous long, thin, heavily branched processes	Thicker main processes with fewer branches	No processes or 1–2 short, thick processes with significantly reduced or non-existing branching

For cytokine assays, the brain tissue (the whole neonatal hemisphere, forebrain hemisphere of young adult) were homogenized 1:3 weight to volume in RIPA buffer (Thermo Scientific) with complete protease inhibitor, followed by centrifugation. Cytokines were analyzed in 100 μl aliquots of tissue homogenates using Rat Inflammation antibody Array G1 (RayBiotech, Inc.) according to the manufacturer’s instructions [32]. Values of the cytokine assay were normalized to protein content of tissue homogenate. The data is presented as a fold change compared to control cohorts (lean or lean-LF).

### Western blotting

Brain homogenates were analyzed by immunoblotting on 8% SDS-PAGE gel with anti-CX3CR1 (Novus, 1:500 dilutions) and anti-β-actin (1;5000), followed by peroxidase-conjugated anti-IgG (Vector Laboratories, Burlingame, CA, 1:5000 dilution). The protein bands were visualized using ECL™ WB Detection kit (Amersham-Pharmacia Biotech) and ChemiDoc MP imaging system (BioRad). Band densities were quantified with the ImageJ program. The data is presented as a fold change compared to control cohorts (lean-LF).

### Cell cultures

Mixed glial cultures were prepared from corteces of newborn CD-1 mice, as described previously [34]. Experiments were performed in low-glucose (5.5 mM) minimal essential medium (MEM), as a control “normoglycemic” (NG), and 16.7 mM glucose was considered as high glucose (HG). Palmitic (0.4 and 1.2 mM) and oleic acids (0.4 and 1.2 mM) were bound to 3% serum albumin bovine (fatty acid free, low endotoxin), and thus, in all conditions, MEM was supplemented with 3% BSA. The glucose and fatty acid concentrations were chosen based on levels detected in healthy vs. GDM and/or T2D patients [35, 36].

### Superoxide, nitric oxide, and cytokine measurements in vitro

Superoxide production was analyzed acutely within a 90-min incubation period with glucose and/or fatty acids with dihydroethidium (dHEt; 1 μM) as described previously [37]. The data is presented as a fold change from baseline dHEt readings normalized to control condition (NG) at the peak time of superoxide production (45–60 min). Nitric oxide (NO) production was measured from cell culture medium with Greiss reagent after 24 h of incubation in experimental conditions as previously described [32]. Cytokines were analyzed in 100 μl aliquots of cell culture medium using Mouse Inflammation antibody Array G1 (RayBiotech, Inc.), detecting ten different pro- and anti-inflammatory cytokines according to the manufacturer’s instruction. Values for NO and cytokine assays were normalized to the protein content of each well. The data is presented as a fold change compared to control levels (NG).

### Statistical analysis

For in vivo studies, the *n* denotes the number of rats in each group, which consisted of a minimum of four different litters. For cell culture studies, the *n* denotes the number of independent experiments, each performed in triplicate. All data are expressed as the mean ± SEM. Microglial morphological changes were evaluated with the Kruskal-Wallis test followed by Dunn’s test for multiple group comparisons. Statistical significance of neonatal data was evaluated by one-way ANOVA followed by the Student-Newman-Keuls. All other data were compared with two-way ANOVA followed by Bonferroni’s post hoc test.

## Results

### Gestational diabetes mellitus and/or postnatal high-fat and sucrose diet induce atypical inattentive behavior and recognition memory impairment in offspring

**Fig. 1:**
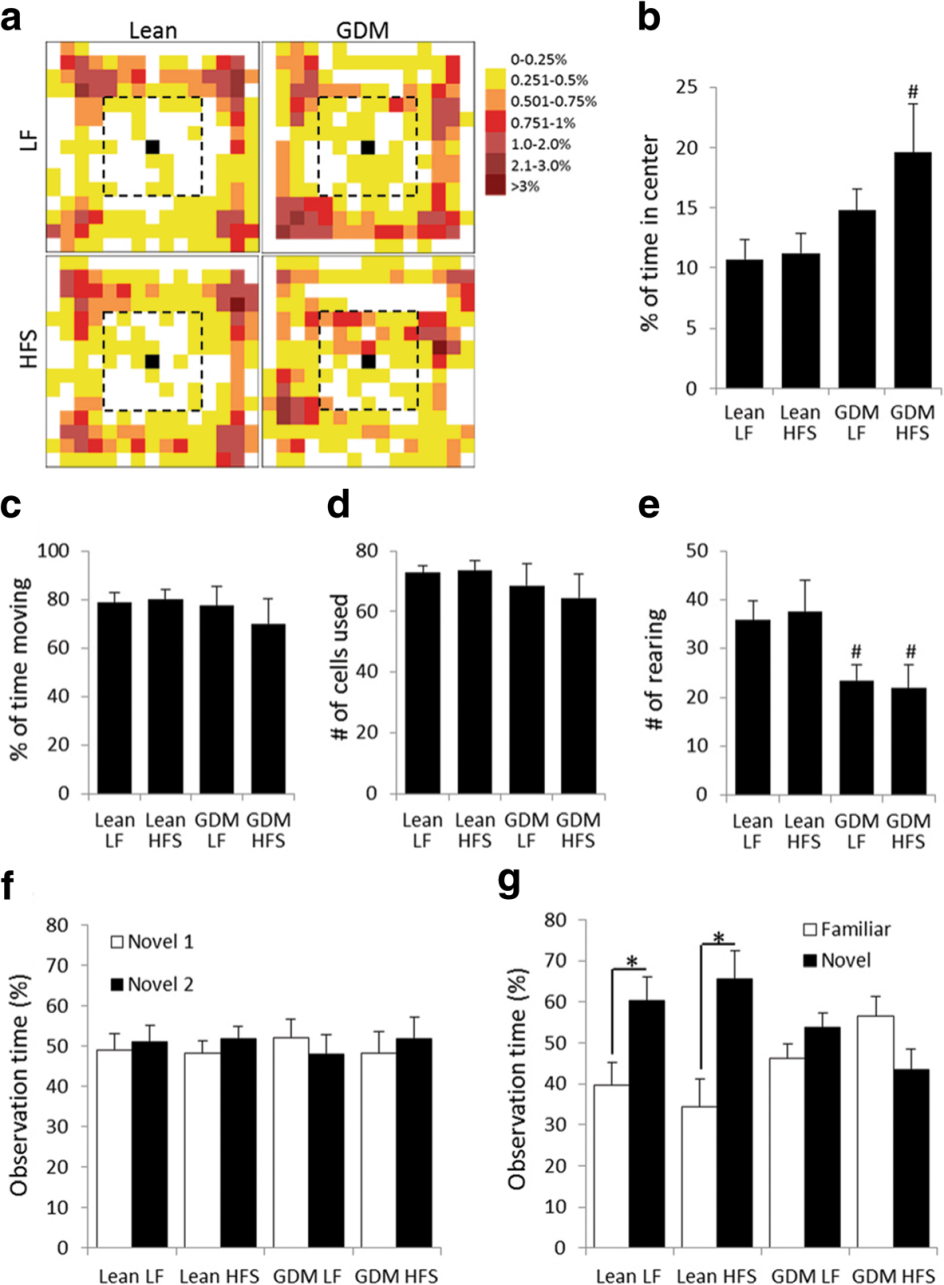
Gestational diabetes mellitus induces atypically exploratory and inattentive behavior and impairs recognition memory in offspring. Behavioral test analysis of 14- to 15-week-old rats. **a**, **b** Open field heat map demonstrates that GDM offspring on postnatal HFS diet stayed more in the center of the field. ^#^ *p* < 0.05, compared to lean LF cohorts, *n* = 8–10. **c**–**e** GDM offspring also showed reduced rearing, but their moving activity was not different from the lean offspring cohorts. **f**, **g** In NOR, GDM offspring failed to differentiate the *familiar object* from the *novel object* presented to them a day after the training, while lean offspring spent significantly more time exploring the *novel object* on test day.*p < 0.05, *n* = 6–10

Offspring of GDM dams were analyzed at 14–15 weeks of age, which is equivalent to the young adult stage in humans. At this age, the GDM offspring are overweight (Table 2) and insulin resistant [25]. General and locomotor activity, as assessed by the OF test, was similar between all groups of offspring (Fig. 1a, c, d). However, significantly fewer rearing were observed by the GDM offspring compared to the lean offspring (Fig. 1e). GDM offspring fed a LF diet (GDM-LF) tended (*p* = 0.054) to spend more time in the center than lean-LF and lean-HFS offspring, while GDM offspring fed a HFS diet (GDM-HFS) spend significantly more (*p* < 0.05) time in the center of the field (Fig. 1b), indicating differences in the exploration habits of the GDM offspring. Rodents typically avoid being exposed to open space but prefer to remain close to the walls as a thigmotactic response [38] and actively observe their surroundings by rearing, suggesting that the GDM-HFS offspring show reduced anxiety level and inattentive behavior as they explore the center of the OF while not actively surveying their surroundings as indicated by their reduced rearing. The NOR test demonstrated that offspring of GDM dams also had impaired recognition memory because they spent equal time exploring both the familiar and novel object, while lean offspring preferred to examine the novel object displaying healthy recognition memory (Fig. 1f, g).

### Gestational diabetes mellitus reduces CA1 neuronal and synaptic integrity in offspring brain

**Fig. 2:**
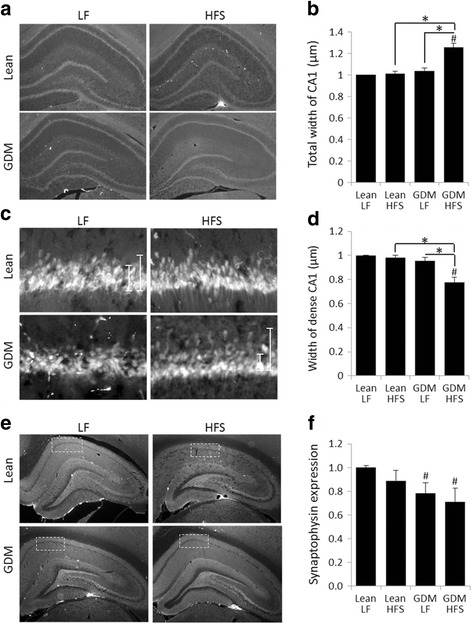
Gestational diabetes mellitus reduces hippocampal synaptic protein expression and deranges CA1 neuronal layer, when combined with postnatal HFS diet. **a**, **b**, **e** Micrographs of 15-week-old rat hippocampus with NeuN and synaptophysin. **a**–**d** NeuN staining did not show clear reduction in neuronal numbers, but changes in CA1 pyramidal neuronal layer thickness and density were evident. **e**, **f** Hippocampal CA1 area in GDM offspring had decreased synaptophysin expression suggesting decreased synaptic terminal integrity. *p ≤ 0.05 compared to lean, *n* = 6

Hippocampal damage is associated with a poor outcome in the NOR test [30, 39]; thus, we performed immunostaining to evaluate hippocampal neuronal and synaptic integrity. A higher overall thickness of NeuN-immunoreactive cells was detected in the neuronal CA1 layer of the GDM-HFS group compared to GDM-LF and lean offspring controls (Fig. 2a, b) due to elevated number of scattered neurons above the organized pyramidal layer. In addition, the compact, tightly connected pyramidal neuron layer was thinner in the GDM-HFS offspring (Fig. 2c, d). Reduced synaptophysin expression was observed in the CA1 and DG area of GDM offspring, compared to offspring of lean dams (Fig. 2e, f). Synaptophysin was also reduced in neonatal GDM offspring by 11.1 ± 3% (*p* = 0.009, *n* = 7) when compared to offspring of lean dams. The CA1 neuronal layer in neonatal offspring could not be delineated as it is still organizing until ~P20 [40, 41]. However, we do not expect changes in neuronal numbers based on the data collected at 15 weeks of age, when only GDM-HFS diet resulted in derangement in the CA1 neuronal layer. This suggests that fetal GDM exposure conditions the cells while the second stress induced by the postnatal HFS diet causes derangement in the CA1 layer.

### Gestational diabetes mellitus induces chronic inflammation in the offspring brain

**Fig. 3:**
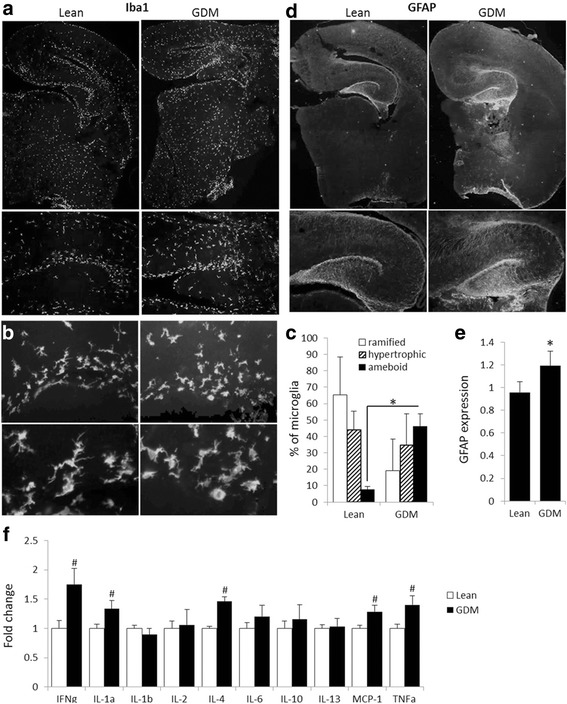
Gestational diabetes mellitus induces neuroinflammation in the offspring brain (at 20E). Micrographs of immunostaining with Iba1 and GFAP demonstrate elevated neuroinflammation status throughout the rat embryo brain. **a**, **b** Iba1 allows visualization of microglial morphology. The microglia of embryos from GDM dams have more activated, amoeboid morphology compared to embryos from dams with healthy pregnancy of which majority possess resting, ramified microglial morphology. **d** Increase in GFAP expression seen in embryos of GDM dams reflects on astroglial activation and chronic inflammation. Quantitative analysis of hippocampal Iba1-positive cells (**c**) and GFAP staining (**e**) confirms the elevated neuroinflammation in GDM offspring. **f** Cytokine analysis demonstrates increase in pro-inflammatory cytokines (IFNγ, TNFα, IL-1, MCP-1), while only IL-4 was elevated from the anti-inflammatory cytokines (IL-4, IL-10, IL-13). *p ≤ 0.05 compared to lean, *n* = 6

Since GDM can promote a pro-inflammatory environment [14], we evaluated activation of microglia, the resident immune cells of the brain. Iba1 immunohistochemical analysis of the hippocampi from neonates revealed microglial morphological activation, as judged by the reduced proportion of ramified microglia (lean 65 ± 11% vs GDM 19 ± 6%), and an increased proportion of microglia exhibiting amoeboid morphology (lean 8 ± 2% vs GDM 46 ± 8%) (Fig. 3a–c). Increased GFAP expression (Fig. 3d, e) in the GDM neonates indicated elevated hippocampal astogliosis. Increased levels of pro-inflammatory cytokines IFNγ, IL-1α, IL-4, MCP-1, and TNFα were also observed in the brain of the neonatal offspring of GDM dams (Fig. 3f). Overall, our findings show that GDM increased neuroinflammation status in the neonatal offspring.

**Fig. 4:**
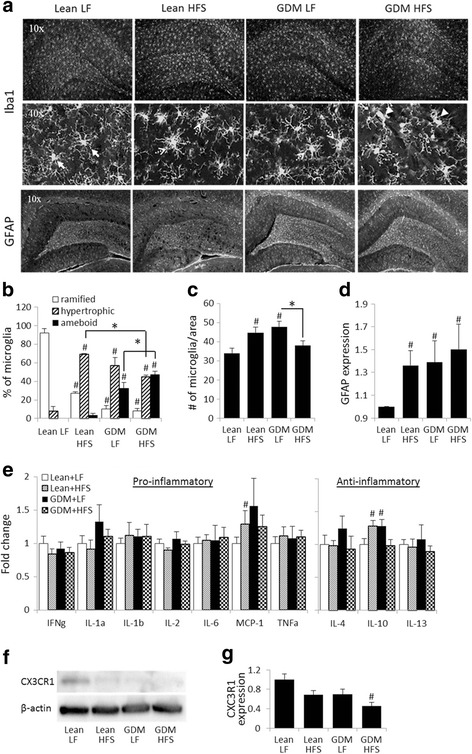
Gestational diabetes mellitus promotes neuroinflammation in offspring (at 15 weeks of age). **a** Micrographs of Iba1 and GFAP immunostainings from 15-week-old rat hippocampi. **b**, **c** Iba1 staining allows visualization of microglial morphology and number of microglia/macrophages present in the hippocampus. Microglia in offspring from lean dams possess resting, ramified morphology, whereas GDM exposure reduced microglial ramification, as evident by increase in hypertrophic morphology and “fully activated” amoeboid morphology in GDM offspring brain. **d** GDM offspring also showed increased GFAP expression which demonstrates astroglial activation upon GDM. **e** Cytokine analysis of 15-week-old rat brain cortical section including the hippocampi, cortex, and hypothalamus does not indicate big changes in cytokine profiles between groups. **f**, **g** CX3CR1 expression was significantly reduced in GDM-HFS offspring brain. ^#^ *p* ≤ 0.05 compared to lean LF, *p ≤ 0.05 as indicated, *n* = 6

The microglial morphological transformation and astrogliosis observed in the GDM offspring persisted into young adulthood (Fig. 4). A reduced proportion of ramified microglia and increased hypertrophic microglia within the hippocampus were observed in the lean-HFS group (Fig. 4a, b). However, both GDM-LF and GDM-HFS groups exhibited reduced ramified microglia and increased hypertrophic and amoeboid microglia indicating neuroinflammation (Fig. 4a, b). Astrogliosis assessed by GFAP staining was elevated in the lean-HFS, GDM-LF, and GDM-HFS offspring groups (Fig. 4a, d). Interestingly, increased hippocampal microglial numbers were observed in the lean-HFS and the GDM-LF groups (32 ± 17.2 and 40 ± 17.7%, respectively), whereas the GDM-HFS group exhibited a 20.4 ± 0.1% reduction in microglial numbers compared to GDM-LF offspring (Fig. 4c). Microglial numbers in the GDM-HFS group were not below the levels observed in lean-LF offspring. Cytokine analysis of the young adults from the various groups did not reveal drastic changes in levels of cytokines in the brain tissue (Fig. 4e). Among pro-inflammatory cytokines measured, only MCP-1 was elevated in the brain of lean-HFS offspring (Fig. 4e), whereas the anti-inflammatory cytokine, IL-10 was increased in the brain of the both lean-HFS and GDM-LF offspring groups (Fig. 4e).

### GDM reduces microglial CX3CR1 levels

Since microglial morphological activation correlates with CA1 neuronal derangement and reduction in synaptophysin expression in the GDM offspring, we evaluated whether GDM affects microglial CX3CR1 expression, which has been suggested to have an important role in regulation of microglial functions associated with resting signals and phagocytosis [42, 43]. While a postnatal HFS diet in lean offspring tended to reduce CX3CR1 expression in the brain (31.6 ± 9.3%, *p* = 0.055), GDM exposure also tended to reduce CX3CR1 expression in LF-fed offspring (30.6 ± 10.5%, *p* = 0.057) and an additive effect of GDM exposure and postnatal HFS diet consumption on CX3CR1 expression was observed in the GDM-HFS offspring (54.6 ± 7.3%, *p* = 0.015) (Fig. 4f, g).

### High glucose and palmitic acid exposure stimulate pro-inflammatory responses in primary microglial cultures

**Fig. 5:**
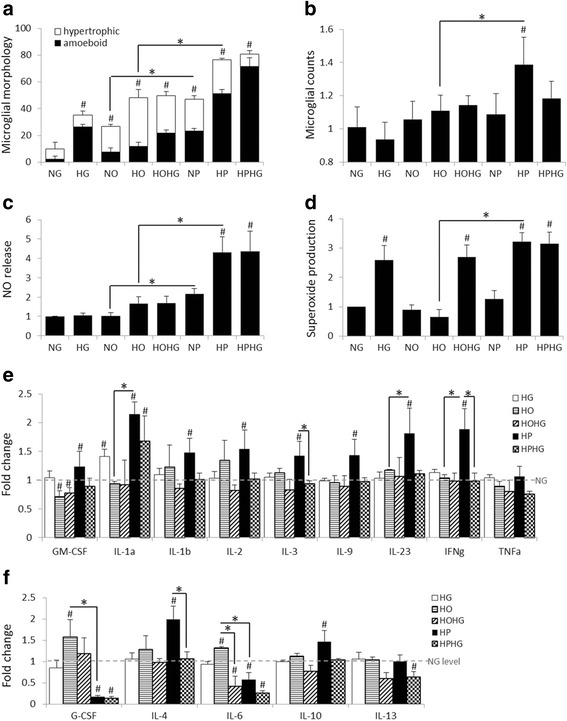
High glucose and palmitic acid exposure stimulates pro-inflammatory response in primary microglial cultures. **a**–**f** Responses of cultured microglia exposed to conditions mimicking T2D; utilizing glucose and fatty acid levels find in T2D vs. healthy patients. High fat (HF; 1.2 mM palmitic acid) alone and with elevated glucose (HG; 16.7 mM) induces microglial transformation towards activated amoeboid morphology (**a**), stimulates microglial proliferation (**b**), triggers release of nitric oxide (NO) (**c**), production of superoxide (SO) (**d**), and alters pro-inflammatory (**e**) and anti-inflammatory (**b**) cytokine release profiles in primary microglial cell cultures. ^#^ *p* ≤ 0.05 compared to control (NG), *p ≤ 0.05 as indicated, *n* = 3-6

To test the hypothesis that GDM conditions and/or individual components of a HFS diet can trigger microglial activation, primary microglia were cultured in elevated glucose and palmitic acid concentrations that are similar to levels observed in the circulation of patients with type 2 diabetes [44, 45]. High glucose (HG) stimulated microglial transformation towards activated morphology (Fig. 5a). Oleic acid, a monounsaturated fatty acid, promoted morphological activation increasing the percentage of hypertrophic microglia, whereas palmitic acid, a saturated fatty acid, increased the percentage of amoeboid microglia (Fig. 5a) and overall microglial numbers (Fig. 5b). Interestingly, the combination of HG and high palmitate (HP) had the most profound effect on increasing the proportion of activated microglia with amoeboid morphology. The combination of oleate and HG did not accentuate microglial morphological activation (Fig. 5a). Microglial NO release was stimulated only by HP and no additive effect was observed with HP and HG (Fig. 5c). Superoxide production was increased by HG or HP, but additive effects of HG and HP were not observed (Fig. 5d). Oleate did not affect NO or superoxide production and the combination of oleate with HG did not potentiate their production (Fig. 5c, d). Microglial cytokine analysis revealed that HG increased IL-1α release (Fig. 5e). Palmitate increased the release of several pro-inflammatory cytokines (GM-CSF, IL-1α, 1β, 2, 3, 9, 23, IFNγ, TNFα) as well as some anti-inflammatory cytokines (IL-4, 10) and reduced IL-6 and G-CSF release (Fig. 5e, f). Oleic acid did not increase pro-inflammatory cytokine release, but stimulated G-CSF and IL-6 release (Fig. 5e, f). Combined HP and HG exposure only increased IL-1α release and reduced IL-6, 13, and G-CSF (Fig. 5e, f). The combination of oleate and HG attenuated the oleate-induced production of IL-6 (Fig. 5f).

## Discussion

Neurons in the CNS require a healthy microenvironment to survive, which is a central function of microglia, the resident immune cells of the brain. In the adult brain, microglia phagocytose invading pathogens and clear away debris to maintain a healthy CNS. During brain development in utero, microglia control the wiring of the brain by performing synaptic pruning and assisting in synapse maturation [23, 46]. Microglial phagocytosis and cytokine release profile also affect neurogenesis and neuronal viability [20, 46]. In obese adult humans and rodents, a prolonged hypercaloric challenge coincides with activation of microglia, which is initially transient but, when sustained, leads to detrimental effects on the surrounding neurons [47].

Our data provides the first evidence that in the rat, maternal obesity-associated GDM influences microglial activation and neuroinflammation in newborn offspring. Moreover, we show for the first time that microglial activation in GDM offspring is sustained into young adulthood and is associated with astrogliosis and derangement of the hippocampal CA1 layer. Interestingly, these alterations in neuronal arrangement in the young adult GDM offspring were associated with impaired recognition memory, reduced anxiety level, and inattentive behavior. Therefore, offspring of GDM dams exhibit many of the cognitive changes reported in some clinical cohort studies of populations of children from mothers diagnosed with diabetes during pregnancy [3–6].

Our study used obesity-associated GDM model, in which dams are fed a diet enrich in high levels of fats and simple sugars. The diet increased the weight of the dams and caused a pregnancy-driven hyperglycemia and hyperinsulinemia, as the glucose intolerance starts after mid-pregnancy [19, 25]. The offspring of our GDM mice have elevated weight gain and, at 15 weeks, show mild insulin resistance, but no glucose intolerance or hyperglycemia [2, 25]. While these symptoms in both dams and offspring are consistent with the clinical presentation of obesity-associated GDM, our GDM rat model does not recapitulate all of the aspects of non-obese GDM that has different etiology [48].

At present, little is known about how excess saturated fats and simple sugars affect microglial activation. Recent research has shown that high-glucose levels increase microglial activation [49] and cytokine production [50, 51]. Similarly, a diet rich in saturated fats [52] as well as intracerebral ventricular injections of saturated fatty acids [53] stimulated microglial activation and inflammatory responses. Previous clinical research [54, 55] and results from experimental mouse models [56] showed that diets high in saturated fat and simple sugars lead to cognitive impairments. Obesity has also reported to induce microglial activation and IL-1R-mediated deficits in hippocampal synaptic plasticity in mice [57]. In agreement with these findings, we observed elevated neuroinflammatory status (microglial morphological activation and astrogliosis) in lean-HFS offspring. We extend these findings to show for the first time that GDM induced by a diet high in saturated fat and simple sugars during pregnancy caused reactive gliosis and elevated pro-inflammatory cytokine (IFN-γ, IL-1α, MCP-1, TNFα) levels in the neonate offspring. Reactive gliosis was maintained until early adulthood; however, inflammatory cytokine levels in the brain were not. Together, this data suggested that fetal exposure to maternal obesity-associated GDM induced robust neuroinflammation in the offspring brain during the critical period of hippocampal synaptic development (up to 3–4 weeks of age). Interestingly, derangement of the CA1 pyramidal neurons and behavioral changes in the GDM offspring were observed only when a postnatal HFS diet was consumed, suggesting that GDM conditions microglia to be in an activated state and the addition of the HFS diet further damages the hippocampal CA1 synaptic organization and maintenance. Previous research using primary microglial cultures showed that raising the glucose concentration from 25 to 50 mM or from 10 to 25 mM increased TNFα secretion two- or [51] fourfold [50], respectively. However, 25 mM glucose is rarely observed in the brain [44]. The glucose levels in adult are about 70–80% lower in CSF than in plasma and do not generally exceed 16.7 mM (300 mg/dl) [58]. However, in neonates and infants, CSF/plasma ratio is higher than in adults 0.88–1.1 [59]. As glucose levels fluctuate due to feeding patterns, we decided to use a 5.5 mM (100 mg/dl) glucose as a normoglycemic condition and significantly higher, 16.7 mM glucose (300 mg/dl) as a high-glucose condition in order to capture the fluctuation maximum in these 24-hour microglial culture experiments in order to assess the effects of gestational glucose exposure. This glucose increase (from 5.5 to 16.7 mM) stimulated morphological activation of microglia without increasing TNFα production, nor other cytokines in general. On the other hand, we observed that elevated palmitate markedly stimulated microglial activation including pro-inflammatory cytokine production, in agreement with previous findings [52]. While the combination of HP and HG triggered superoxide production and morphological activation, it only increased IL-1α cytokine release, whereas HP alone elevated most of the measured pro-inflammatory cytokines. The combination of HP and HG maintained anti-inflammatory cytokines at basal levels (IL-4 and IL-10) or below (IL-13), while HP alone increased IL-4 and 10 levels. While the fold changes in cytokine levels in our study were not profound, the singular cytokine levels may not be as crucial as the sum of the changes in the whole cytokine profile. Based on these findings, elevated saturated fatty acid concentrations appeared to contribute a larger effect on microglial activation and neuroinflammation than elevated glucose levels. This is not surprising given that microglia express the toll-like receptor [53] as well as lipoprotein lipase and acyl-CoA synthases that facilitate fatty acid uptake by cells [51]. Nonetheless, additional studies are required to elucidate the effects of glucose and fatty acid supplementation on microglial cytokine production.

While our study provides strong evidence that microglial actions promote the pathological changes in the hippocampus of GDM offspring, it is important to keep in mind that these changes are likely multifactorial and represent a combination of elevated maternal glucose, fatty acids, and inflammation/cytokines. In fact, a HFS diet has been reported to reduce hippocampal neuronal viability via affecting microvascular insulin sensitivity [60] and fatty acids can be directly neurotoxic [61, 62].

Hippocampal microglial numbers were increased in lean-HFS as well as GDM-LF offspring, while the combination of GDM exposure and a HFS postnatal diet reduced microglial cell numbers. One explanation for this observation is that the HFS diet promotes GDM induced chronic microglial activation to a level where chronic pro-inflammatory activation jeopardized microglial viability. It has been postulated that microglial senescence and lack of microglial supportive functions could lead to neurodegeneration associated with various neurodegenerative diseases [63]. Cytokine analysis of the young adults partially supports this notion, since IL-10, 4, and 13 levels were reduced in the GDM-HFS offspring compared to the GDM-LF offspring.

An additional explanation for the cognitive deficits seen in young adult GDM offspring was reduced levels of synaptic proteins and a drastic decrease in microglial expression of the fractalkine receptor, CX3CR1. The fractalkine (CX3CL1)-CX3CR1 signaling axis promotes microglial remodeling of synaptic circuits by regulating microglial motility, trophic factor release, and phagocytosis activity [43]. Microglial CX3CR1 expression in the developing CNS is essential for hippocampal synapse development [23], synaptic plasticity, and cognitive functions [64]. In neurodegenerative disease models, CX3CR1 depletion was reported to sustain microglial inflammatory responses and degrade synaptic circuits, thus promoting neurodegeneration [65]. Our in vivo findings are in line with these findings and reduced CX3CR1 expression provides a logical mechanistic explanation linking microglial changes to hippocampal synaptic degradation, neuronal derangement, and cognitive changes in GDM offspring. A recent report further supports the view that prenatal stress can program microglial CX3CR1 expression and promote microglial pro-inflammatory responses [66]. Given that neuroinflammation is largely driven by microglia, and our data shows that GDM causes microglial activation that is associated with hippocampal neuronal weakening and cognitive deficits, a reduction in microglial CX3CR1 expression could be central in the sustained microglial activation observed in the 15-week-old offspring of GDM dams.

## Conclusions

Our findings suggested that obesity-associated GDM induces neuroinflammation in offspring during the critical period of hippocampal synaptic development dramatically affecting hippocampal neuronal synaptic integrity and cognitive abilities. GDM exposure combined with postnatal HFS feeding to the offspring induced further hippocampal damage as indicated by the derangement of hippocampal pyramidal neurons in the CA1 area. Mechanistically, postnatal HFS diets and prenatal GDM exposure induced reduced CX3CR1 expression that could explain impaired microglial functions, prolonged pro-inflammatory microglial responses, and uncontrolled synaptic phagocytosis. Therefore, microglial activation in the fetal brain could serve as an adaptive response to elevated fatty acids and glucose during a GDM pregnancy, which appears to condition microglia to a neuroinflammatory environment such that sustained exposure to postnatal HFS diet resulted in derangement of the hippocampal CA1 pyramidal neurons and cognitive impairments in young adulthood. Understanding the mechanisms responsible for the effects of GDM on cognitive development in the offspring could provide new ways to intervene and thus modify the neurodevelopmental effects of GDM.

## Abbreviations

CA1: Cornu Ammonis 1
CNS: Central nervous system
DG: Dentate gyrus
G-CSF: Granulocyte colony-stimulating factor
GDM: Gestational diabetes mellitus
GM-CSF: Granulocyte macrophage colony-stimulating factor
HFS: High fat and sucrose
HG: High glucose
HP: High palmitate
IFNγ: Interferon gamma
IL: Interleukin
LF: Low fat
MCP-1: Monocyte chemoattractant protein-1
NG: Normoglycemic
NO: Nitric oxide
NOR: Novel object recognition
OF: Open field
TNFα: Tumor necrosis factor alpha
